# Bio-organic fertilizers modulate the rhizosphere bacterial community to improve plant yield in reclaimed soil

**DOI:** 10.3389/fpls.2025.1660229

**Published:** 2025-09-16

**Authors:** Cece Qiao, Jing Yang, Qingqin Shao, Jingli Fu, Xueyun Zheng, Jianrong Zhao, Lantian Ren, Wenge Wu, Jianfei Wang

**Affiliations:** 1 College of Resource and Environment, Anhui Science and Technology University/Anhui Engineering Research Center for Smart Crop Planting and Processing Technology, Chuzhou, China; 2 Rice Research Institute, Anhui Academy of Agricultural Sciences, Hefei, China

**Keywords:** reclaimed soil, bio-organic fertilizer, microbial community, plant yield, MiSeq platform 1

## Abstract

Soil reclamation is a crucial strategy for restoring degraded land and improving agricultural productivity, yet the underlying microbiological mechanisms that drive soil quality improvement remain inadequately understood. To address this, a rice field experiment under chemical fertilizer (CF), organic fertilizer (OF), and *B. subtilis*-enriched bio-organic fertilizer (BOF) was conducted to assess the impact of different fertilization treatments on rhizosphere soil bacterial community by targeted sequencing in reclaimed paddy soil. The results revealed that BOF significantly enhanced rice yield and improved soil attributes, including a reduction in soil pH and an increase in microbial diversity. Compared with the CF and OF, BOF exhibited a more pronounced effect on the enrichment of indigenous *Bosea* spp. in the rhizosphere. Metabolomic analysis further revealed that the relative abundance of *Bosea* was positively associated with increased levels of aromatic compounds such as *benzoic acid* and *tropolone*, which are potentially linked to improved soil functionality. These findings suggest that the synergistic interaction between *B. subtilis* and native *Bosea* populations may enhance soil health and promote sustainable crop productivity by altering microbial community structure and activating beneficial metabolic pathways. Collectively, this study provides further insight into the role of bio-organic fertilizers in promoting ecological restoration and sustainable agriculture in reclaimed soils.

## 1 Introduction

With the increasing scarcity of global arable land resources, the agricultural reuse of reclaimed soil has become an important way to ensure food security. Concurrently, the reclaimed soil area has been exceeded 7.3 million hectares and continues to increase at a rate of 3.2% every year with the rapid urbanization process in China (Li et al., 2024). Reclaimed soil refers to the regenerated soil formed through systematic restoration of damaged land caused by mining subsidence, engineering construction, and urbanization processes through artificial intervention measures. However, reclaimed soil generally suffers from prominent problems such as nutrient deficiency, pH imbalances and relative lower microbial diversity index, resulting in rice yield hovering only at 40-60% namely 2.8 t ha^-1^ of normal cultivated land (Mola et al., 2024). In this case, China has been confronted urgent need of land reclamation, mainly involving improvement of soil fertility and improved biomass productivity.

Although traditional physico-chemical methods could temporarily stabilize soil pH and available nutrients, it still indicated difficulty to rebuild stable soil microecological systems. To solve these concerns, bioremediation method has been applied to improve nutrient utilization and maintain pH balance, directly improving agroecosystem productivity based on soil microorganisms in reclaimed soils (Liang et al., 2022). As the largest biodiversity of agroecosystems, the soil microbiome performs fundamental functions such as promotion of nutrient utilization, decomposition of organic matter, regulating plant health and maintaining soil structure, and thus directly determines soil fertility and ecological functions in agroecosystems (Sokol et al., 2022). To date, increasing evidences highlight the critical role of rhizospheric soil microorganisms where plant roots and microbial interactions in remediation (Bi et al., 2021; Yu et al., 2024). For example, the rhizospheric microbial consortia *Rhizobium* species could improve nitrogen fixation efficiency in soybeans grown on acidic reclaimed soils (Bhunia et al., 2021). Current research has well identified and optimized the rhizosphere’s core microbial groups responsible for regulating acidification and nutrient dynamics in conventional soil (Li et al., 2025). These findings broadened implementation of microbiome-based strategies for soil restoration. However, most researches has been focused on traditional conventional soil based on long-term field experiments (Huang et al., 2024). Farmers intend economic benefits from amended reclaimed soil within a shorter time frame from the perspective of practical application. Thus, given the key role of microbial communities in regulating soil function, a better mechanistic understanding how they respond to different fertilization schemes would therefore contribute to improve soil quality and enhance crop productivity in reclaimed soil.

The over-reliance on chemical fertilizers has exacerbated the degradation of reclaimed soils, causing further disruption to microbial diversity and nutrient cycling, thus impairing ecological stability and reducing agricultural productivity (Li et al., 2021). In response to these challenges, bio-organic fertilizers have emerged as a promising alternative. It could enhance nutrient cycling by introducing functional microbial consortia and restructure the soil microbiome, significantly improving soil health and crop yields (Yang et al., 2025). As one of the key microbial agents for bio-organic fertilizers, *Bacillus* is known for its adaptability to diverse soil environments. *Bacillus* facilitates nutrient transformation and stabilizes organic matter, making it a vital component of soil amelioration strategies (Liu et al., 2023). Furthermore, recent studies have shown that bio-organic fertilizer applications lead to the excretion of organic acids, increasing bio-available phosphorus concentrations by 40-60% in reclaimed soils (Liu et al., 2024; Duan et al., 2023). Although bio-organic fertilizer have shown potential in improving soil fertility, their ability in reclaimed soil to reshape bacterial community and selectively enrich specific bacterial populations crucial for enhancing soil rehabilitation and optimizing crop productivity remains unexplored (Jin et al., 2022; Li et al., 2021). Thus, further research is needed to understand how *Bacillus-*enriched bio-organic fertilizer impact microbial community composition and functionality as well as the soil productivity.

Given the importance of functional microorganisms in soil restoration, a field experiment was conducted with three different fertilization treatments: chemical fertilizer (CF), organic fertilizer (OF), and bio-organic fertilizer (BOF) to investigate how the application of bio-organic fertilizer influences soil fertility, microbial diversity, and crop productivity in reclaimed soils. The overall specific objectives were to: 1) evaluate the effects of *B. subtilis*-based bio-organic fertilizer on plant yield in reclaimed soils; 2) decipher how BOF affect the structural and functional dynamics of rhizospheric soil bacterial communities; and 3) identify specific key bacterial taxa associated with soil properties and crop yields. This study would contribute to develop more efficient and sustainable fertilizer management strategies for grain production in reclaimed soils.

## 2 Materials and methods

### 2.1 Site overview and experimental design

This experimental site was originally a rural residential area and had been converted into clay loam soil approximately five years before the study. Then the field experiment has been conducted in He County, Maanshan City, Anhui Province, China (31°76′80″N, 118°30′07″E) for four years, demonstrating that the B. subtilisbased bio-organic fertilizer has a significant ability to promote plant growth. It has a temperate climate, and the average temperature during the rice planting days was 24.8°C. Baseline soil characteristics indicated poor fertility, with a pH of 7.29, organic matter (SOM) of 7.54 g kg^-1^, total N, P and K (TN, TP, TK) were at 0.85 g kg^-1^, 0.65 g kg^-1^, 4.18 g kg^-1^, available P and K (AP, AK) were at 9.90 mg kg^-1^, and 36.00 mg kg^-1^, respectively.

**Figure 1:**
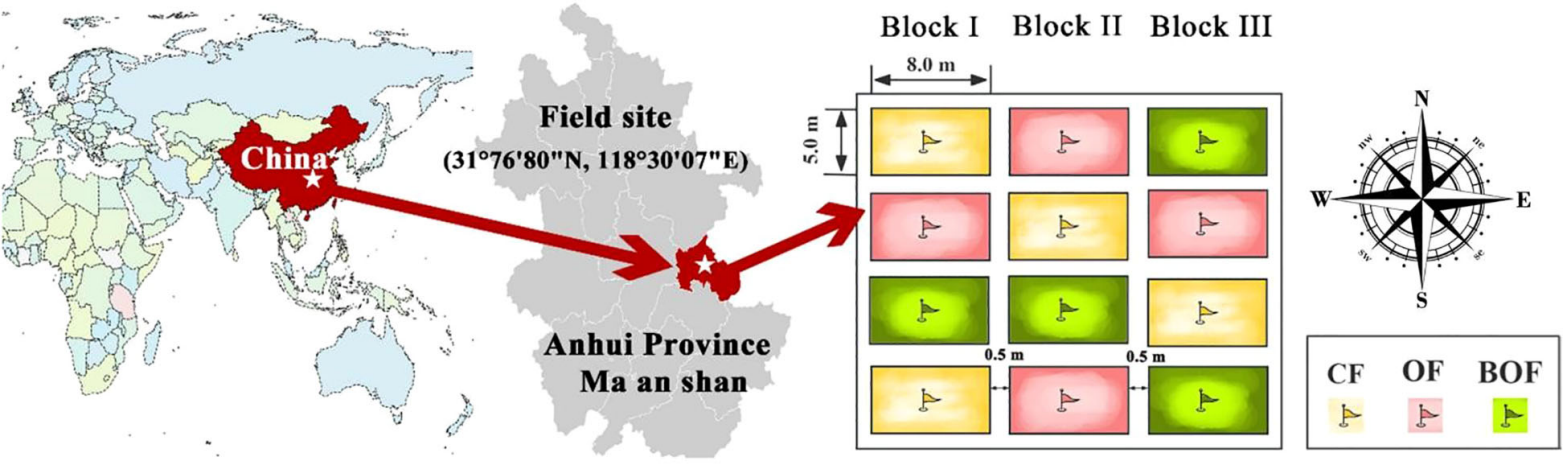
Experimental site and layout plan of the residential area.

A randomized complete block design in four replicates with three treatments was employed (**Figure 1**), including: (i) chemical fertilizer (CF), (ii) organic fertilizer (OF), and (iii) bio-organic fertilizer (BOF) containing *B. subtilis*. The chemical fertilizer included nitrogen (N), phosphorus pentoxide (P_2_O_5_), and potassium sulfate (K_2_SO_4_). The organic fertilizer was derived from mushroom compost by maintaining composting temperature for 7 days above 55 °C, containing 360 g kg^-1^ organic matter, 16.8 g kg^-1^ N, 12.5 g kg^-1^ P_2_O_5_, and 10.3 g kg^-1^ K_2_O with a pH value of 7.87. Mix *Bacillus* powder directly into the decomposed organic fertilizer at a ratio of 0.5-2% (i.e. adding 5–20 kg of bacterial agent per ton of organic fertilizer). As such, the BOF was prepared by inoculating *B. subtilis* at a concentration of 4.3 × 10^7^ CFU g^-1^ (dry weight). OF and BOF treatments received an organic fertilizer application rate of 220 kg per 667 m², while the CF treatment received only chemical fertilizer. All plots were supplied with equivalent total amounts of nitrogen (240 kg ha^-1^), phosphorus (125 kg ha^-1^), and potassium (180 kg ha^-1^). 50% of organic and chemical fertilizers were applied as a basal dose before transplanting, followed by 30% at the jointing stage and 20% at the heading stage. Rice was transplanted in June 2024 and harvested in October 2024. During the harvest stage, each plot was collected individually. Then the rice yield was calculated based the dry weight of each plot.

### 2.2 Soil collection, properties characterization, and DNA isolation

Following the harvest, six rhizosphere subsamples were collected from each replicate plot and pooled to form one composite sample for every treatment. Rhizosphere soil sampling was conducted following the protocol described by Qiao et al. (2019), with minor modifications. Briefly, rice roots were gently shaken to remove loosely adhering soil. The tightly attached soil was dislodged by washing the roots with sterile saline solution. Then the resulting suspension was centrifuged at 10,000 rpm for 10 minutes, and the sediment was collected as rhizosphere soil. All rhizosphere soil samples were immediately stored at -70°C for further DNA isolation.

The other collected soil from each composite sample was airdried for further physicochemical analysis. All detected methods followed standardized protocols outlined in Qiao et al. (2019).

DNA extraction was made from 0.20 g of frozen rhizosphere soil and the chloroplast blocker primers were used. Then the quality and concentration of DNA were tested.

### 2.3 PCR amplification and 16S rRNA gene sequencing

The bacterial 16S rRNA gene was amplified using the universal premes 341F (5’-ACTCCTACGGGAGGCAGCAG-3’) and 806R (5’-GGACTACHVGGGTWTCTAAT-3’) (Klindworth et al., 2013). Each 25 mL PCR reaction mixture contained 0.15 U mL^-1^ Taq polymerase, 450 mM dNTPs, 15 mM Tris-HCl (pH 8.0), 100 mM KCl, and 3 mM MgCl2, 0.8 mL (10.5 mM), 1.0 mL of template DNA, and nuclease-free water 9.9 mL. Purified amplicons were sequenced using the Illumina MiSeq platform.

The sequencing data were uploaded to NCBI with the BioProject number PRJNA1271970.

### 2.4 Bioinformatics and data statistical analysis

Raw 16S rRNA gene sequences were processed using the QIIME2 pipeline (version 2021.4) for subsequent analysis. Low-quality reads, and chimeric sequences were filtered out using DADA2. Both forward and reverse reads were merged to ensure consistent sequencing quality. Primer sequences were trimmed prior to denoising, enabling consistent orientation of the reads. Amplicon sequence variants (ASVs) were inferred, and taxonomic classification was carried out using the RDP Classifier against the SILVA 138 database with a confidence threshold of 70%.

All statistical analyses were performed using R (version 4.5.1) and IBM SPSS Statistics 26.0. Data normality was assessed using the Shapiro-Wilk test before parametric testing. Depending on the experimental design, one-way analysis of variance (ANOVA) after checking the homoscedasticity or independent sample t-tests were used to evaluate treatment effects, with statistical significance was *P* < 0.05.

Depending on the experimental design, ANOVA after checking the homoscedasticity or independent sample t-tests were used to evaluate treatment effects, with the significance P < 0.05. An ASV-based analysis was applied to calculate the alpha diversity of the bacterial community richness (Chao index) and diversity (Shannon index). PCoA were applied to visualize bacterial community structure differences across fertilization treatments. The significant differences in microbial community composition were determined by PERMANOVA in R (version 4.5.1). Linear analysis was conducted to identify significantly different taxa between treatments. Spearman’s rank correlation assessed associations between ASV abundance and agronomic traits such as rice yield. The Mantel test examined relationships between bacterial community dissimilarities and soil physicochemical variables.

## 3 Results

### 3.1 Effects of fertilization treatments on rice yield

**Figure 2:**
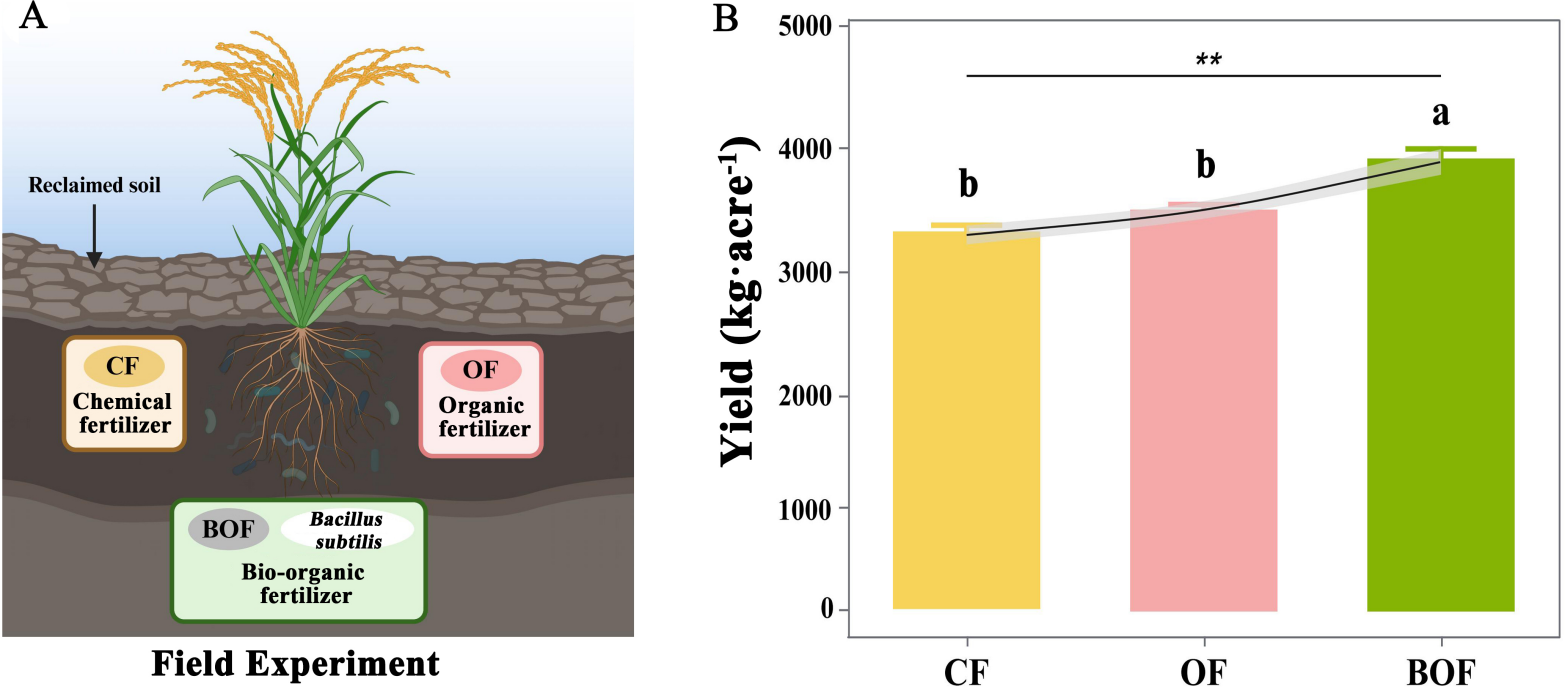
The influence of different fertilization treatments (CF, OF, BOF) on rice yields in reclaimed soils. **(A)** Fertilizer treatments: chemical fertilizer (CF), organic fertilizer (OF), and bio-organic fertilizer (BOF) with *B*. *subtilis*. **(B)** Rice yields (mean ± SE) under each treatment based on the number of replicate plots (n = 4); ANOVA followed by Duncan’s test (*P*<0.05) **P < 0.01.

In comparison to organic (OF) and chemical (CF) fertilizers (**Figure 2A**), the application of bio-organic fertilizer (BOF) significantly increased rice yield by 413 kg acre^−1^ and 591 kg acre^−1^, respectively (Duncan’s test, *P* < 0.05; **Figure 2B**). In contrast, the difference between CF and OF treatments was not statistically significant (*P* > 0.05) in reclaimed soils. The significant yield improvement under BOF compared to OF (*P* < 0.05) emphasizes the efficacy of BOF as a more beneficial fertilization approach for boosting rice productivity in reclaimed soils. These findings demonstrated the superior yield performance of bio-organic fertilizer with *B. subtilis*, supporting its potential as a viable and effective solution for enhancing rice productivity in soils with nutrient deficiencies.

### 3.2 Effects of soil physical and chemical properties

Compared to the conventional CF, both of the organic amendments (OF and BOF) significantly improved soil pH (**Table 1**), indicating the effect of alleviating acidification associated with BOF application. The BOF significantly increased the SOM (40.2 g kg^-1^) concentrations by 69.5% when compared to CF but not OF. As for the available nutrient stuffs, the BOF (27.26 mg kg^-1^) significantly improved the AP by 13.39% and 9.46% than CF (23.61 mg kg^-1^) and OF (24.68 mg kg^-1^), respectively. In particular, no significant difference was observed respected to AK of the three fertilization treatments.

**Table 1:** Soil physicochemical properties under different fertilization treatments.

Fertilizer treatment)	pH	SOM (g kg^-1^)	Available P (mg kg^-1^)	Available K (mg kg^-1^)	Total N (g kg^-1^)	Total P (g kg^-1^)	Total K (g kg^-1^)
CF	7.13 ± 0.14c	8.79 ± 0.48b	23.61 ± 0.56c	41.67 ± 10.21b	1.21 ± 0.08b	0.60 ± 0.06b	7.32 ± 0.16b
OF	7.38 ± 0.08b	10.71 ± 0.60a	24.68 ± 0.26b	46.00 ± 3.95b	1.36 ± 0.04a	0.64 ± 0.05b	7.91 ± 0.23a
BOF	7.65 ± 0.06a	11.12 ± 0.65a	27.26 ± 0.6a	58.67 ± 3.26a	1.37 ± 0.07a	0.76 ± 0.03a	7.95 ± 0.04a

Physicochemical characteristics of soil across varying fertilizer applications. Data represent mean values ± standard deviation. Lowercase letters (a, b, c) denote statistically significant variations among treatments (one-way ANOVA, *P* < 0.05), while “NS” signifies no notable differences. Corresponding *P*-values from the ANOVA for each soil parameter are included.

As for the total nutrient stuffs, the application of bio-organic fertilizer resulted in significantly higher TN, TP and TK as compared to CF, while no significant differences were found in TN and TK levels between BOF and OF. Additionally, BOF (0.76 g kg^-1^) revealed a clearly increased TP compared to CF (0.60 g kg^-1^) and OF (0.64 g kg^-1^). Overall, These findings collectively suggested that BOF application improves key nutrient levels and induces mild changes in soil pH, potentially enhancing nutrient availability and microbial activity.

### 3.3 Impacts of bacterial community diversity and composition

**Figure 3:**
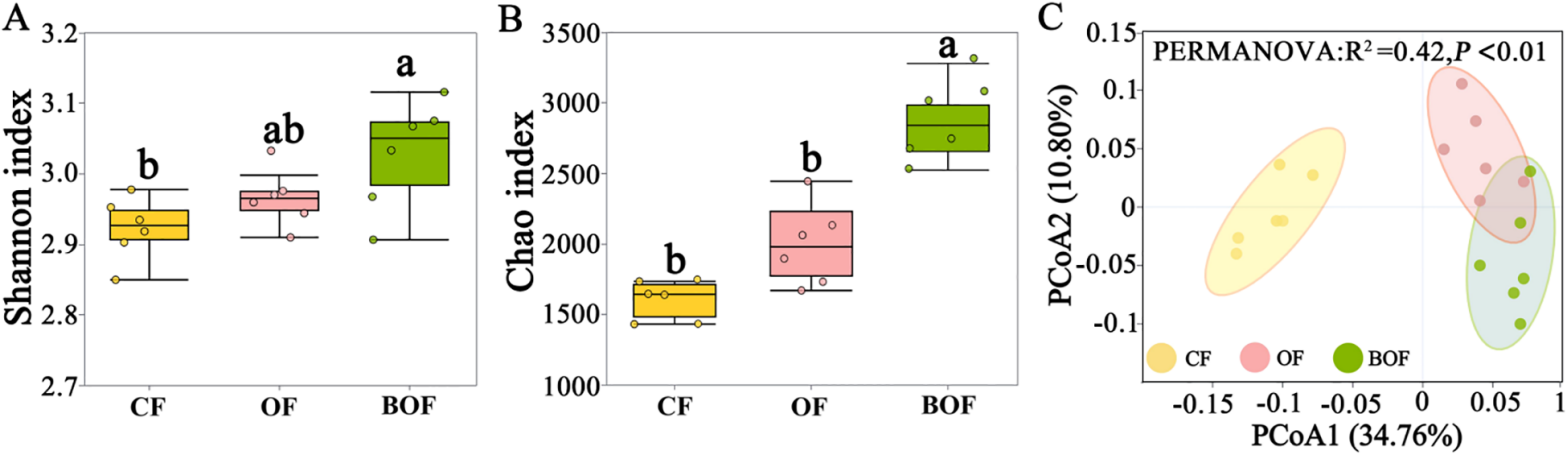
Bio-organic fertilizer (BOF) reshaped the rhizosphere bacterial community. **(A)** Bacterial α-diversity indices Shannon value under different treatments. **(B)** Bacterial α-diversity indices Chao value, the x-axis labels means fertilizer treatments and the lowercase letters indicate significance in **(A, B)**. **(C)** PCoA based on Bray-Curtis dissimilarity showing community composition across treatments.

The α-diversity of the bacterial community was evaluated using the Shannon and Chao indices to assess richness and diversity under different fertilization treatments (**Figures 3A, B**). Compared to CF, the application of bio-organic fertilizer significantly increased both indices, indicating improved bacterial richness and diversity. Furthermore, BOF exhibited significantly higher diversity metrics than OF but not richness. For β-diversity, the bacterial communities formed distinct clusters based on fertilization type (**Figure 3C**), with clear separation observed between the CF group and the organic treatments (OF and BOF). This pattern was further validated by permutational multivariate analysis of variance (PERMANOVA), which revealed a significant effect of fertilizer treatment on community composition (*P* < 0.001, R² =0.42, F = 24.91). These results indicated that BOF application could promote bacterial diversity and substantially alter the bacterial community composition.

### 3.4 Identification of responsive bacterial taxa

**Figure 4:**
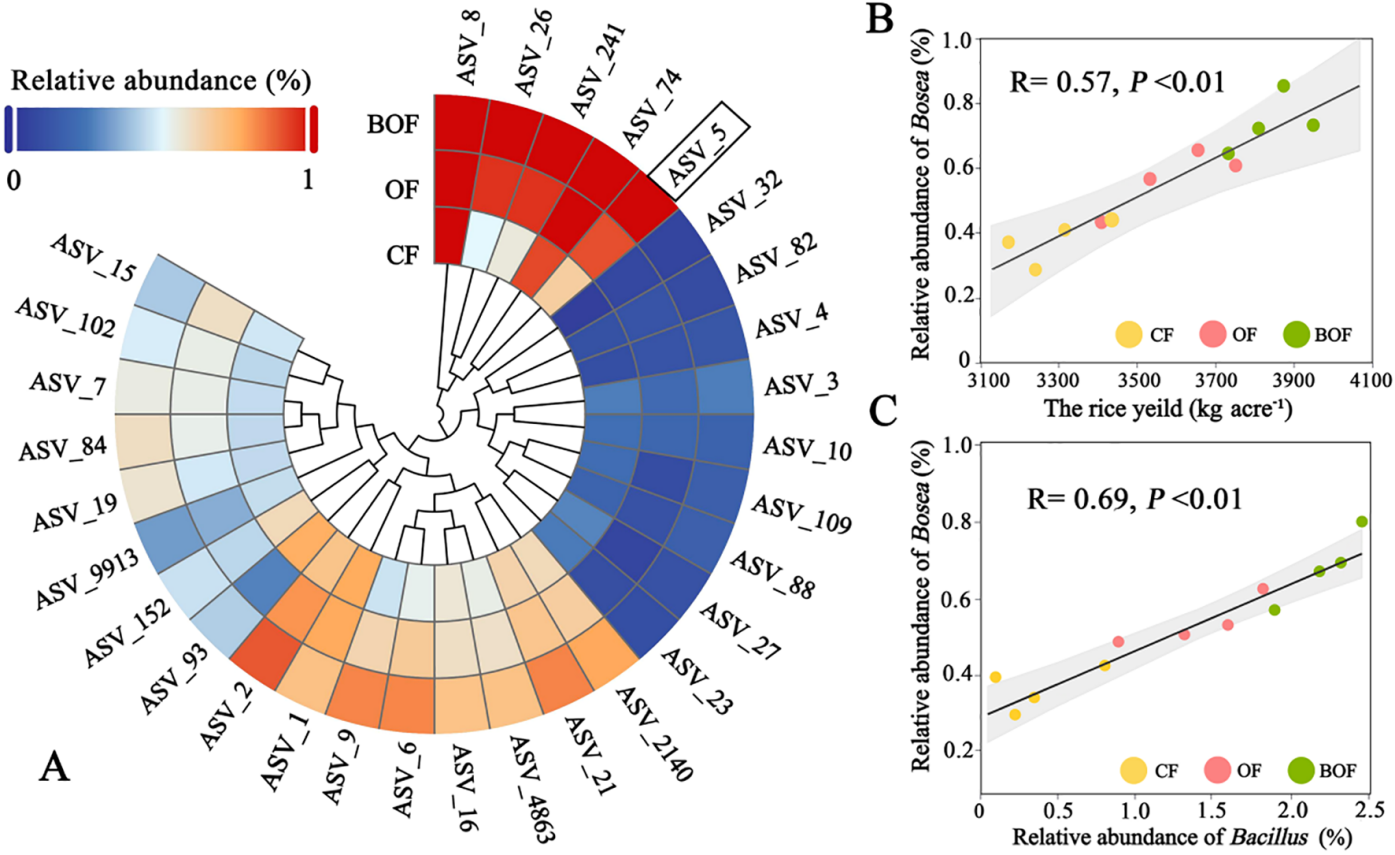
Identification of responsive bacterial taxa. **(A)** Heatmap of responsive bacterial ASV abundance under different treatments by hierarchical clustering. **(B)** Linear regression between *Bosea* abundance and rice yield. **(C)** Correlation between *Bacillus* and *Bosea* relative abundance across treatments, based on linear regression.

The bacterial taxa associated with fertilization strategies and yield enhancement were identified by analyzing bacterial ASVs that exhibited considerable variation across treatments (**Figure 4A**). Seven hundred distinct rhizosphere ASVs showed significant differences among the three fertilizer groups. Spearman’s rank correlation analysis revealed that 30 ASVs positively correlated with rice yield (false discovery rate, FDR < 0.05). Among which, ASV-5 classified as *Bosea* was observed the highest correlated. In the BOF treatment, ASV-5 accounted for 0.71% of the total bacterial community, which was significantly higher than its relative abundance in CF (0.49%) and OF (0.26%) treatments (*P* < 0.05, Duncan’s test). This substantial enrichment of *Bosea* under BOF treatment prompted further investigation into its potential role in enhancing crop yield. Spearman correlation coefficients between the abundances of *Bosea* and soil productivity further confirmed *Bosea* could be key biomarker taxon enriched under BOF treatment, highlighting its potential involvement in nutrient cycling and plant-microbe interactions (**Figure 4B**). Besides, a strong positive correlation was observed between the abundances of *Bosea* and *Bacillus* in the rhizosphere (Spearman’s r = 0.69, *P* < 0.01; **Figure 4C**). Therefore, it was reasonable to speculate that the functional bacteria of bio-organic fertilizers could induce key bacterial consortia to promote crop yield.

**Figure 5:**
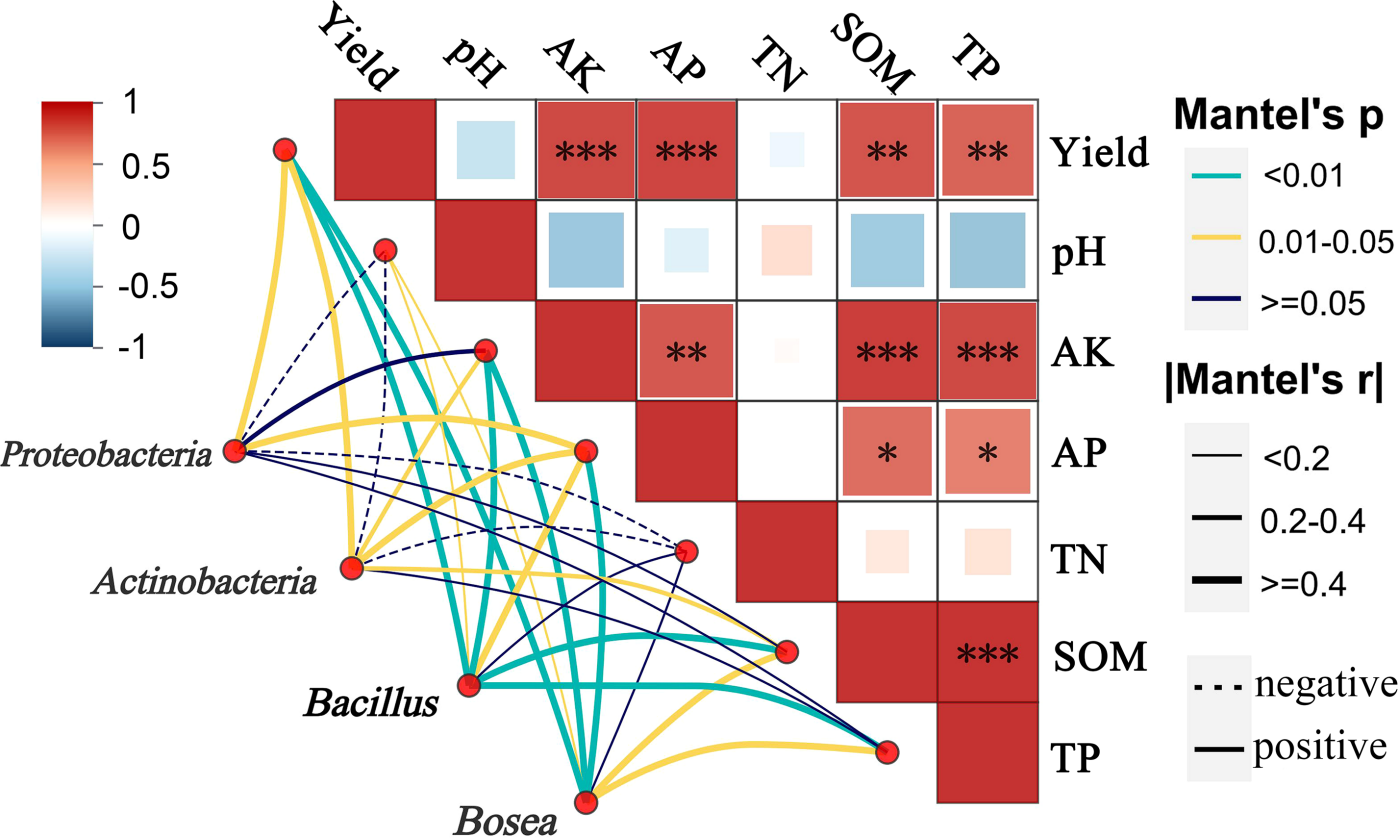
Mantel test correlations between key bacterial taxa (*Proteobacteria*, *Actinobacteria*, *Bacillus*, and *Bosea*) and soil properties, including pH, available potassium (AK), available phosphorus (AP), total nitrogen (TN), total phosphorus (TP), soil organic matter (SOM). Edge styles indicate correlation direction (solid meas positive, dashed means negative), and Mantel’s r and *P*-values are shown *means the correlations of significance at (P<0.05); ** (P<0.01); *** (P<0.001).

Additionally, mantel test correlations between key bacterial taxa and soil properties indicated both the functional microorganisms *Bacillus* and *Bosea* positively correlated with pH, AK, TP and SOM (**Figure 5**), revealing the critical role of bio-organic fertilizers in mitigating soil acidification, increasing organic matter, and activating soil nutrients. These findings suggest that *Bosea* and *Bacillus* work synergistically under BOF treatment, enhancing soil nutrient profiles and promoting rhizosphere adaptation, thereby supporting improved crop productivity.

### 3.5 Synergistic effects of rhizosphere bacterial communities and metabolite enrichment

**Figure 6:**
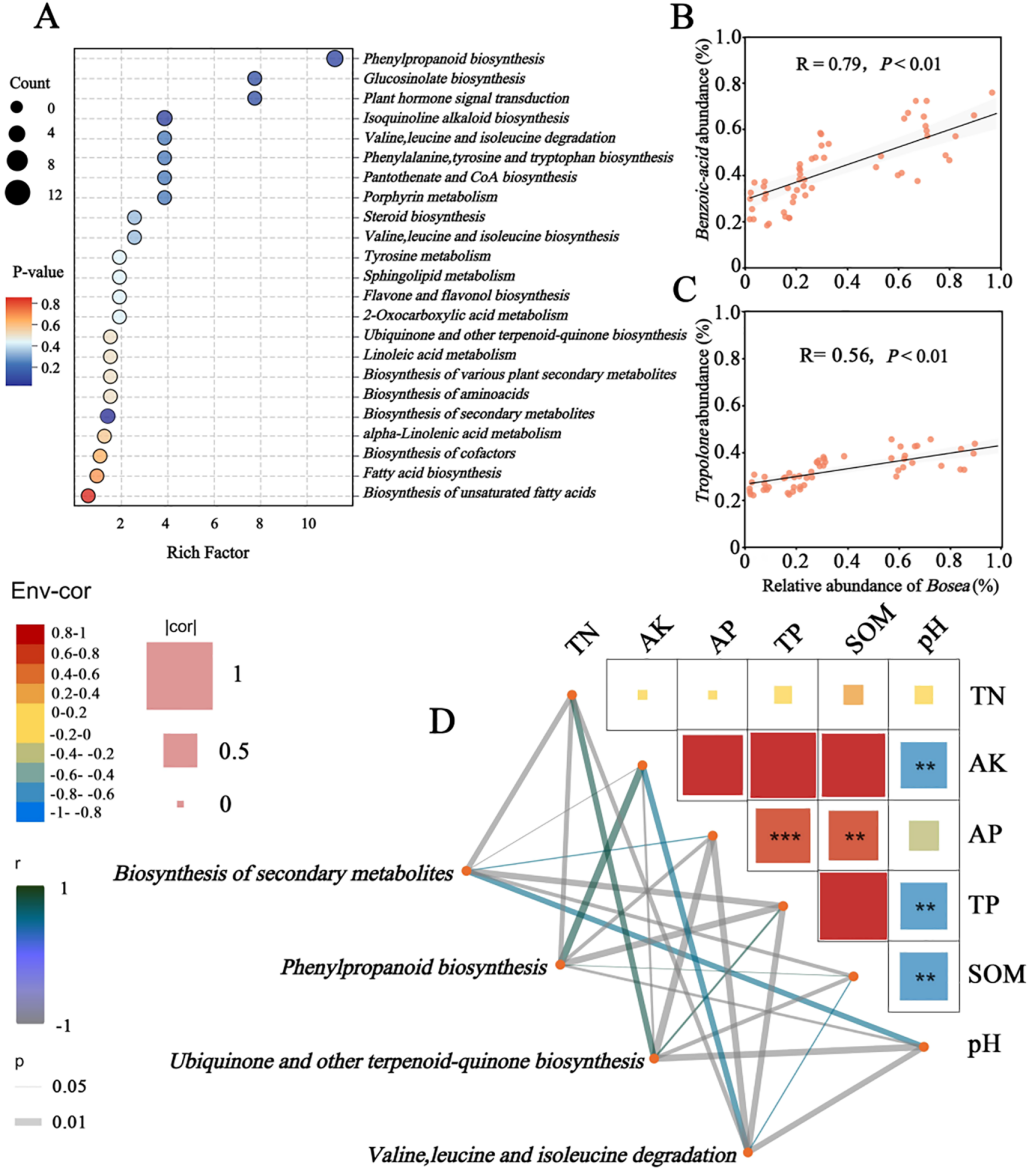
Associations between microbial taxa, metabolic pathways, and soil properties under different fertilization treatments. **(A)** Bubble plot of pathway enrichment under different treatments. Bubble size reflects the number of pathways, and color denotes statistical significance (P-value). **(B)** Linear regression between *Bosea* abundance and benzoic acid concentration across treatments. **(C)** Linear regression between *Bosea* abundance and tropolone concentration across treatments. **(D)** Mantel correlations between key soil properties and enriched metabolic pathways. Edge color represents positive correlations, and dashed lines indicate negative correlations (Mantel r and *P*-values are shown) *means the correlations of significance at (P<0.05); ** (P<0.01); *** (P<0.001).

Rhizosphere metabolite profiles and their correlations with microbial abundance and soil properties were analyzed to investigate the potential metabolic mechanisms underlying the synergistic interaction between *Bosea* and *B. subtilis* under bio-organic fertilizer (BOF) treatment. Notably, the concentrations of tropolone and benzoic acid were highest in BOF soils (Duncan’s test, *P* < 0.05; **Figure 6A**). Regression analysis revealed strong positive correlations between benzoic acid and *Bosea* (r = 0.79, *P* < 0.01, **Figure 6B**), as well as between tropolone and *Bosea* abundance (r = 0.56, *P* < 0.01, **Figure 6C**). These results suggest that *Bosea* proliferation under BOF treatment was likely associated with the accumulation of specific root-associated metabolites. Functional pathway analysis indicated increased activity of phenylpropanoid biosynthesis and secondary metabolite production pathways in the rhizosphere soils of BOF (Kruskal-Wallis test, *P* < 0.05; **Supplementary Figure S2**). These enriched pathways were significantly correlated with key soil properties such as AK (r = 0.64), AP (r = 0.81), SOC (r = 0.64), and SOM (r = 0.62) (**Figure 6D**), indicating that the amendment of bio-organic fertilizers could induce variations in bacterial community function, further activating soil nutrients and increasing soil fertility.

## 4 Discussion

### 4.1 The impact of fertilization treatments on rice yield and soil properties in reclaimed soil

It has been a consensus that bio-organic fertilizers can improve soil function and crop productivity in various ways mainly including optimizing soil organic matter and structure (Jin et al., 2022), activating microbial communities and functions (Tang et al., 2025), reducing pollution and ensure safety (Tao et al., 2020). Recent studies have emphasized that organic fertilizers alone are often insufficient to restore degraded soils while improved plant productivity combined with beneficial microorganisms based on traditional basic farmland (Du et al., 2022; Huang et al., 2023). Accordingly, the present study demonstrated that BOF significantly enhanced rice yield and soil fertility especially the available nutrients in reclaimed soil. This conclusion was derived consistently from Wang et al. (2024), who emphasized the yield promoting impact of bio-organic fertilizer based on reclaimed land. Here, the improvement of yield is considered to be mainly attribute to an enhanced soil fertility triggered by the amendment of bio-organic fertilizer (Lyu et al., 2023). Simultaneously, further soil index should be considered including N mineralization, phosphate solubilization or enzyme activities to substantiate the enhanced nutrient cycling. Considering the relatively lower fertility of reclaimed soil, bio-organic fertilizer can rapidly reconstruct the aggregate structure and microbial community of reclaimed soil by supplementing organic matter and beneficial microorganisms, significantly improving water and fertilizer retention capacity, and thus improved rice yield in the claimed soil. This finding underscore the potential of bio-organic fertilizers in rehabilitating nutrient-poor soils by enhancing rice yield.

### 4.2 Effects of bio-organic fertilizer on rhizosphere bacterial communities in reclaimed soil

The present findings supported that the amendment of bio-organic fertilizer could increase the richness and diversity of claimed soil compared to CF alone. This may be attributed to the application of organic matter in bio-organic fertilizer, which provides more suitable nutritional conditions for the growth and reproduction of microorganisms, in comparison to chemical fertilizers (Tong et al., 2025). In addition, although there was no significant difference in richness between the BOF and OF amendments, there was also an increasing trend, suggesting that the application of functional bacterium *B. subtilis* may have induced more microorganisms. Therefore, the application of bio-organic fertilizers may play an critical role in maintaining different claimed soil rhizosphere bacterial communities and corresponding activities (Pan et al., 2025). Furthermore, the fertilization mode were considered to be the main factors affecting the composition of reclaimed rhizosphere soil bacterial communities, determined by PCoA and PERMANOVA. Previous studies compared the effects of applying chemical, organic, bio-organic fertilizers and found that fertilization was an important factor in reshaping the rhizosphere soil microbial community (Sun et al., 2015). In essence, it was a process of remodeling rhizosphere microecology through multifaceted biological pathways. Primarily, the functional microorganism *Bacillus* indicated strong nutritional and spatial competition abilities, and could rapidly reproduce in the rhizosphere and occupy ecological niches, thereby remodeling microbial community structure (Deng et al., 2022). Besides, *Bacillus* could activate plant systemic resistance (ISR) and change the composition of root exudates, thus indirectly affects the microbial community (Yang et al., 2023). As mentioned above, the application of organic fertilizers has a cumulative effect on the formation of organic matter in rhizosphere soil, thereby promoting the development of rhizosphere microbial communities with higher microbial diversity. Gu et al. (2009) also suggested that the changes in bacterial community composition were more pronounced in rhizosphere soil treated with organic fertilizers than chemical fertilizers. The results of the present study indicated that bio-organic fertilizer could provide more effective nutrients (such as ammonium nitrogen, available phosphorus, and available potassium) to promote the proliferation of microbial growth including beneficial bacteria.

### 4.3 The bio-organic fertilizer induced beneficially responsive rhizosphere bacterial taxa in reclaimed soil

The various bacterial genus *Bacillus* strains have been enhanced within the rice rhizosphere and contributed to the promotion of plant growth (Mahapatra et al., 2022). In the present study, the BOF signifcantly induced the relative abundances of *Bacillus* in comparison to OF and CF, and highlighted the significant role of *B. subtilis* under BOF treatment in contributing to the observed increase in rice yield in reclaimed soil. This result likely stems from the amendment of bio-organic fertilizer containing *B. subtilis*, which directly stimulated soil microbial communities that are essential for nutrient cycling. This was mainly due to *Bacillus* could enhance nutrient solubilization (Liu et al., 2021) and promote the development of root-associated microbial networks (Xia et al., 2025).

However, the relative abundance of *Bacillus* was not as high as we expected, thus indicating that there were the indigenous populations of beneficial microbes. Of which, the positive correlations between the relative abundance of *Bosea*, key soil nutrient parameters, and rice yield suggested that this beneficial genus acts as a keystone species in bio-organic fertilized soils, playing a pivotal role in nutrient cycling and plant growth promotion. The bacterial genus *Bosea* has been reported to be indicated tobacco growth-promoting activities (Yuan et al., 2022) and was involved nitrogen fixation (Zheng et al., 2025). Besides, Khanal et al. (2025) reported the *Bosea* strain participate in assimilatory nitrogen metabolic pathway associated enzymes such as nitrate reductase, which could be the reason that the BOF harbored the highest content of total N in the present study. Recent research has further emphasized the critical role of key microbial taxa in promoting plant health and soil productivity, particularly in the context of reclaimed land amendment strategies (Tabardillo et al., 2025). These findings support the hypothesis that BOF had a multifaceted impact on soil productivity by directly introducing plant-growth-promoting bacteria and indirectly enhancing the diversity and activity of native microbial communities, and thus improved soil function, particularly in reclaimed soils. In the present study, our analysis is mainly based on theoretical calculations and the conclusion is also inferential. Subsequently, it is necessary to isolate the dominant ASV-5 *Bosea* strain and validate the effect of plant promoting growth.

Notably, the abundance of the native bacterial genus *Bosea* was substantially enriched in BOF, suggesting a potential synergistic interaction between introduced and indigenous microbes in driving these beneficial outcomes. While the precise molecular mechanisms remain to be fully elucidated (Giri et al., 2020), our data strongly suggest that the co-enrichment of *B. subtilis* and *Bosea* leads to enhanced plant growth through various mechanisms, such as improved microbial activity in rhizosphere colonization, and the production of beneficial metabolites (Li et al., 2024). The positive correlation between *Bosea* abundance and increased soil nutrient availability including available phosphorus, organic matter, and other key nutrients-further, supporting the hypothesis that microbial interactions improve soil nutrient cycling. Additionally, the reduced soil pH observed under BOF treatments likely created a more favorable environment for nutrient uptake and microbial activity, which is crucial for plant growth.

### 4.4 Potential mechanisms underlying microbial interactions and rice yield improvement

Metabolomic profiling provides additional evidence for the functional role of these microbial interactions. The reclaimed soils in BOF exhibited elevated levels of tropolone and benzoic acid, metabolites which were known to influence microbial behavior and modulate rhizosphere signaling (Orozco-Mosqueda et al., 2021). The significant association between these metabolites and the abundance of *Bosea* suggested a metabolic crosstalk between introduced and native microbes, possibly underlying their co-enrichment (Rong et al., 2021). This metabolic interaction could contribute to the enhanced microbial functionality and plant growth observed in BOF soils (Mitra et al., 2023). Concurrently, the enrichment of biosynthetic pathways, such as phenylpropanoid metabolism, indicates that BOF may alter soil biochemical conditions in ways that favor beneficial microbial colonization and strengthen plant-microbe interactions, thus improving soil health and crop yield. Future work will prioritize the origin of functional pathway phenyl-propanoid metabolites to further verify the conclusion.

**Figure 7:**
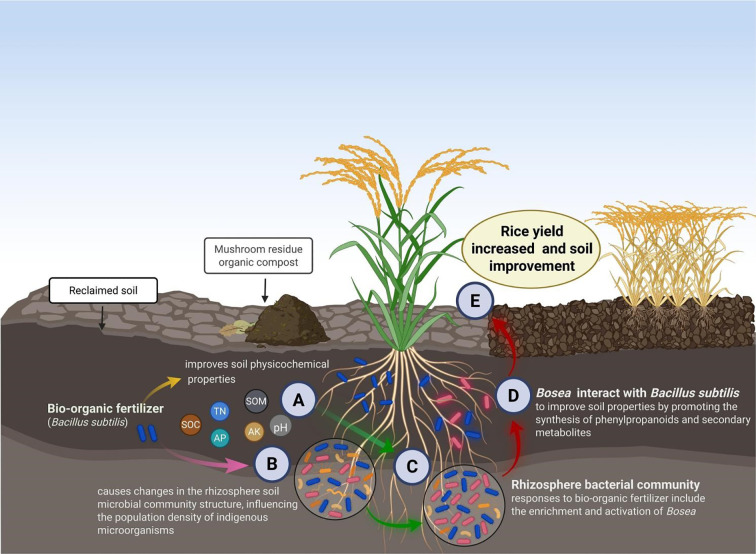
Conceptual framework illustrating how *B*. *subtilis*-enriched bio-organic fertilizer (BOF) improves rice yield and rhizosphere health. **(A)** Adjustment of soil physicochemical conditions, including increased soil organic matter (SOM), enhanced nutrient availability, and pH optimization. **(B)** Restructuring of microbial communities, with selective enrichment of *Bosea*. **(C)** Activation of indigenous plant-beneficial microorganisms in the rhizosphere. **(D)** Induction of phenylpropanoid biosynthesis and secondary metabolite pathways. **(E)** Enhanced root-zone environment and plant productivity.

These findings support the hypothesis that *B. subtilis*-based bio-organic fertilizers promote the plant growth not only by facilitating nutrient availability, reshaping rhizosphere microbial networks, but also through activating functional metabolites (**Figure 7**). This integrated mechanism could be particularly beneficial for rehabilitating nutrient-poor and structurally degraded soils, where the soil microbiome is crucial in restoring fertility (McKinley, 2019). Further research by multi-omics approaches including metagenomics and metabolomics is essential to dissect the specific roles of microbial interactions in improving soil function and plant productivity across different crops and agroecological systems.

While *Bosea* was strongly associated with improved soil nutrient status and increased rice yield, its precise functional contributions remain to be experimentally validated. Future studies should focus on isolating representative strains of *Bosea* and *B. subtilis*, and exploring their individual and combined roles in nutrient transformation and plant growth promotion using metagenomic and GeoChip assay on composite samples to obtain further functional evidence.

## 5 Conclusions

In conclusion, this study demonstrates that *B. subtilis*-enriched bio-organic fertilizer (BOF) significantly enhances rice productivity and soil health in rehabilitated farmland. The conceptual model (**Figure 7**) illustrates how BOF application reshaped bacterial and metabolic transformations that led to enhanced crop productivity and soil functionality: (A) improvement in soil physicochemical properties, such as reduced pH, increased organic matter, and enhanced nutrient availability; (B) restructuring of the rhizosphere bacterial community, characterized by an increase in the abundance of beneficial taxa; (C) activation of native plant-growth-promoting microorganisms such as *Bosea*; and (D) stimulation of phenylpropanoid and secondary metabolite biosynthesis, facilitating rhizosphere conditioning. These synergistic effects collectively contributed to improved rice yield and enhanced soil quality.

Our findings highlight the importance of both inoculated and indigenous microorganisms in driving BOF effectiveness and emphasize the critical role of *Bosea* as a responsive taxon under microbial-enriched fertilization. The study lays the foundation for developing targeted microbial strategies to enhance soil functionality, particularly in degraded or reclaimed agricultural systems. Future applications may benefit from co-inoculation strategies that combine functionally complementary microbial species. Therefore, bio-organic fertilizers should be selected based on nutrient content and the capacity to enrich beneficial microbial networks, offering a pathway toward more sustainable and microbially informed agricultural management.

## Data Availability

The datasets presented in this study can be found in online repositories. The names of the repository/repositories and accession number(s) can be found in the article/**Supplementary Material**.
