# Correction: Bio-organic fertilizers modulate the rhizosphere bacterial community to improve plant yield in reclaimed soil

**DOI:** 10.3389/fpls.2025.1709443

**Published:** 2025-11-27

**Authors:** Cece Qiao, Jing Yang, Qingqin Shao, Jingli Fu, Xueyun Zheng, Jianrong Zhao, Lantian Ren, Wenge Wu, Jianfei Wang

**Affiliations:** 1 College of Resource and Environment, Anhui Science and Technology University/Anhui Engineering Research Center for Smart Crop Planting and Processing Technology, Chuzhou, China; 2 Rice Research Institute, Anhui Academy of Agricultural Sciences, Hefei, China

**Keywords:** reclaimed soil, bio-organic fertilizer, microbial community, plant yield, MiSeq platform 1

The order of authors in the author list of the published paper was originally:

“Cece Qiao ^1^, Qingqin Shao ^1^, Jingli Fu ^1^, Xueyun Zheng ^1^, Jianrong Zhao ^1^, Lantian Ren ^1 *^, Jing Yang ^1 *^, Wenge Wu ^1,2^ and Jianfei Wang ^1^”

The corrected author list reads:

“Cece Qiao^1^, Jing Yang^1*^, Qingqin Shao^1^, Jingli Fu^1^, Xueyun Zheng^1^, Jianrong Zhao^1^, Lantian Ren^1*^, Wenge Wu^1,2^, Jianfei Wang^1^”.

A correction has been made to the title. The original title “Bio-organic fertilizers modulate therhizosphere bacterial community to improve plant yield in reclaimed soil” has been corrected to “Bio-organic fertilizers modulate the rhizosphere bacterial community to improve plant yield in reclaimed soil.”

There were some errors in section **2.Materials and methods** - *2.1 Site overview and experimental layout*. The original paragraph stated:

“This experimental site was originally a rural residential area and had been converted into clay loam soil approximately five years before the study. Then the field experiment has been conducted in He County, Maanshan City, Anhui Province, China (31°76′80″N, 118°30′07″E) for four years, demonstrating that the *B. subtilis*-based bio-organic fertilizer has a a significant ability to promote plant growth. The region has a temperate monsoon climate with an average annual temperature of 15.8 °C. Baseline soil characteristics indicated poor fertility, with a pH of 7.29, organic matter content of 7.54 g kg-1, total nitrogen at 0.85 g kg-1, total phosphorus at 0.73 g kg-1, total potassium at 4.18 g kg-1, available phosphorus at 9.90 mg kg-1, and available potassium at 36.00 mg kg-1.”

A correction has been made to the section:

“This experimental site was originally a rural residential area and had been converted into clay loam soil approximately five years before the study. Then the field experiment has been conducted in He County, Maanshan City, Anhui Province, China (31°76′80″N, 118°30′07″E) for four years, demonstrating that the *B. subtilis*-based bio-organic fertilizer has a significant ability to promote plant growth. It has a temperate climate, and the average temperature during the rice planting days was 24.8°C. Baseline soil characteristics indicated poor fertility, with a pH of 7.29, organic matter (SOM) of 7.54 g kg^-1^, total N, P and K (TN, TP, TK) were at 0.85 g kg^-1^, 0.65 g kg^-1^, 4.18 g kg^-1^, available P and K (AP, AK) were at 9.90 mg kg^-1^, and 36.00 mg kg^-1^, respectively.”

There were some errors in section *2.2 Soil collection, properties characterization, and DNA isolation*. The original paragraph stated:

“The other collected soil from each composite sample was airdried for physicochemical analysis including pH, soil organic carbon (SOC), total nitrogen (TN), total phosphorus (TP), total potassium (TK), available phosphorus (AP), available potassium (AK). All detected methods followed standardized protocols outlined in Qiao et al. (2019). Genomic DNA was extracted from 0.20 g of frozen rhizosphere soil using the MoBio PowerSoil DNA Isolation Kit (QIAGEN, USA) and the chloroplast blocker primers were used. The quality and concentration of extracted DNA were evaluated using a NanoDrop spectrophotometer (Thermo Fisher Scientific, USA).”

A correction has been made to the section:

“The other collected soil from each composite sample was air-dried for further physicochemical analysis. All detected methods followed standardized protocols outlined in Qiao et al. (2019).

DNA extraction was made from 0.20 g of frozen rhizosphere soil and the chloroplast blocker primers were used. Then the quality and concentration of DNA were tested.”

There were some errors in section *2.3 PCR amplification and 16S rRNA gene sequencing*, Paragraphs 1 and 2. The original section stated:

“The V3-V4 hypervariable region of the bacterial 16S rRNA gene was amplified using the universal premes 341F (5’-ACTCCTACGGGAGGCAGCAG-3’) and 806R (5’-GGACTACHVGGGTWTCTAAT-3’) (Klindworth et al., 2013). Each 25 mL PCR reaction mixture contained 12.5 mL of 2×Taq PCR Master Mix (containing 0.15 U mL-1 Taq polymerase, 450 mM dNTPs, 15 mM Tris-HCl (pH 8.0), 100 mMKCl, and 3 mMMgCl2), 0.8 mL of each primer (10.5 mM), 1.0 mL of template DNA, and 9.9 mL of nuclease-free water. The thermal cycling conditions were as follows: initial denaturation at 95°C for 5min; followed by 30 cycles of denaturation at 95°C for 30 s, annealing at 55°C for 30 s, and extension at 72°C for 45 s; with a final extension at 72 °C for 10 min. PCR products were confirmed by 1.5% agarose gel electrophoresis and purified using the AxyPrep DNA Gel Extraction Kit (Axygen, USA). Purified amplicons were sequenced using the Illumina MiSeq platform (2 × 250 bp paired-end) by a commercial service provider (Majorbio Bio-Pharm Technology Co., Ltd., Shanghai, China).

Raw data have been deposited to National Center for Biotechnology Information (NCBI) under the BioProject number PRJNA1271970.”

A correction has been made to the section:

“The bacterial 16S rRNA gene was amplified using the universal premes 341F (5’-ACTCCTACGGGAGGCAGCAG-3’) and 806R (5’-GGACTACHVGGGTWTCTAAT-3’) (Klindworth et al., 2013). Each 25 μL PCR reaction mixture contained 0.15 U μL^-1^ Taq polymerase, 450 μM dNTPs, 15 mM Tris-HCl (pH 8.0), 100 mM KCl, and 3 mM MgCl2, 0.8 μL (10.5 μM), 1.0 μL of template DNA, and nuclease-free water 9.9 μL. Purified amplicons were sequenced using the Illumina MiSeq platform.

The sequencing data were uploaded to NCBI with the BioProject number PRJNA1271970.”

There were some errors in section **2 Materials and methods**, *2.4 Bioinformatics and data statistical analysis*, Paragraphs 2 and 3. The original section stated:

“An ASV-based analysis was performed to calculate the alpha diversity of the bacterial community richness (Chao index) and diversity (Shannon index). Principal coordinate analysis (PCoA) based on bray-Curtis dissimilarity metrics were applied to visualize bacterial community structure differences across fertilization treatments. Permutational multivariate analysis of variance (PERMANOVA) was conducted using the “adonis” function in R’s vegan package (version 4.5.1) to determine significant differences in microbial community composition. Linear analysis based on Spearman correlation coefficients was conducted to identify significantly different taxa between treatments. Spearman’s rank correlation assessed associations between ASV abundance and agronomic traits such as rice yield. The Mantel test examined relationships between bacterial community dissimilarities and soil physicochemical variables.”

A correction has been made to the section:

“Depending on the experimental design, ANOVA after checking the homoscedasticity or independent sample t-tests were used to evaluate treatment effects, with the significance P < 0.05.

An ASV-based analysis was applied to calculate the alpha diversity of the bacterial community richness (Chao index) and diversity (Shannon index). PCoA were applied to visualize bacterial community structure differences across fertilization treatments. The significant differences in microbial community composition were determined by PERMANOVA in R (version 4.5.1). Linear analysis was conducted to identify significantly different taxa between treatments. Spearman’s rank correlation assessed associations between ASV abundance and agronomic traits such as rice yield. The Mantel test examined relationships between bacterial community dissimilarities and soil physicochemical variables.”

There was an error in **Figure 2**, where “Yield” and “Field” were misspelled as “Yeild” and “Feild”. There was an error in **Figure 5** where “Yield” was misspelled as “Yeild”. The corrected figures are displayed here.

**Figure 2:**
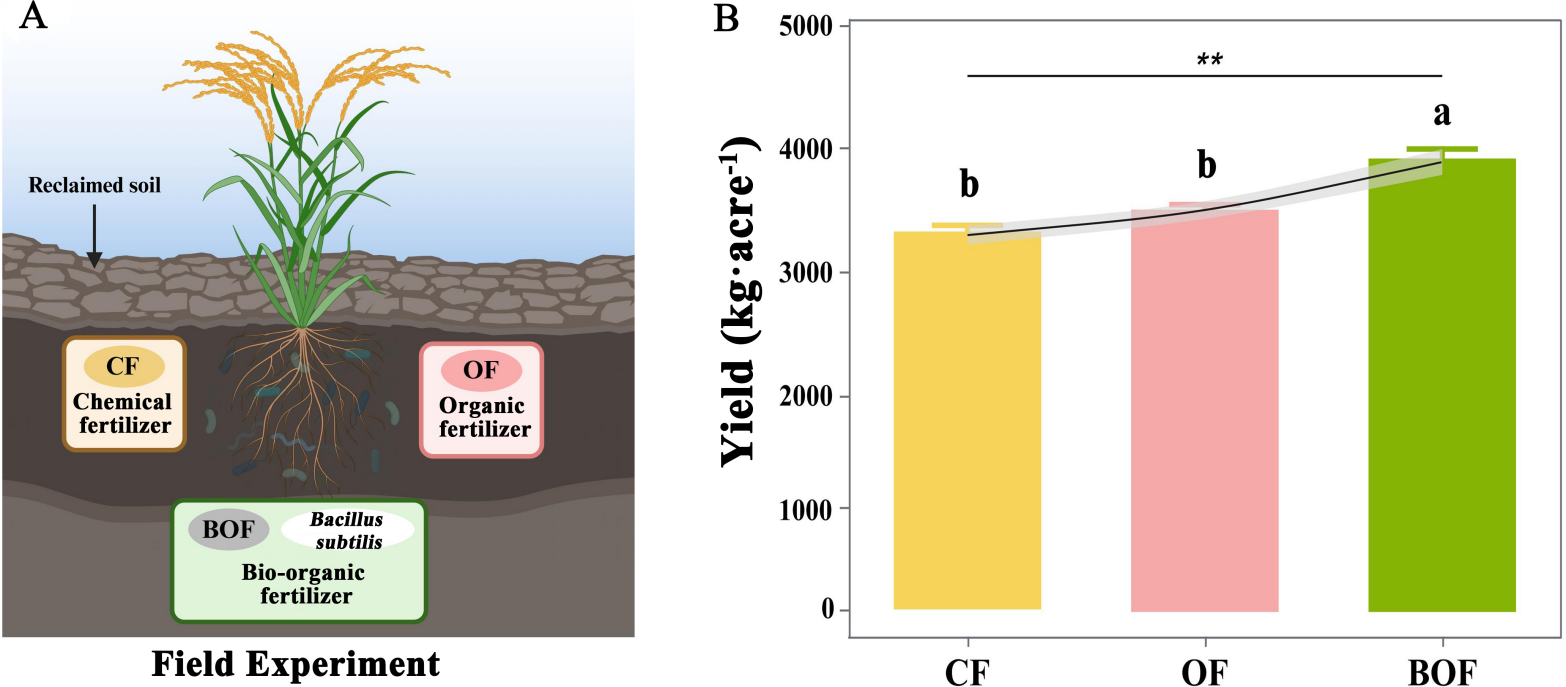
The influence of different fertilization treatments (CF, OF, BOF) on rice yields in reclaimed soils. **(A)** Fertilizer treatments: chemical fertilizer (CF), organic fertilizer (OF), and bio-organic fertilizer (BOF) with *B. subtilis*. **(B)** Rice yields (mean ± SE) under each treatment based on the number of replicate plots (n = 4); ANOVA followed by Duncan’s test (*P*<0.05) **P < 0.01.

**Figure 5:**
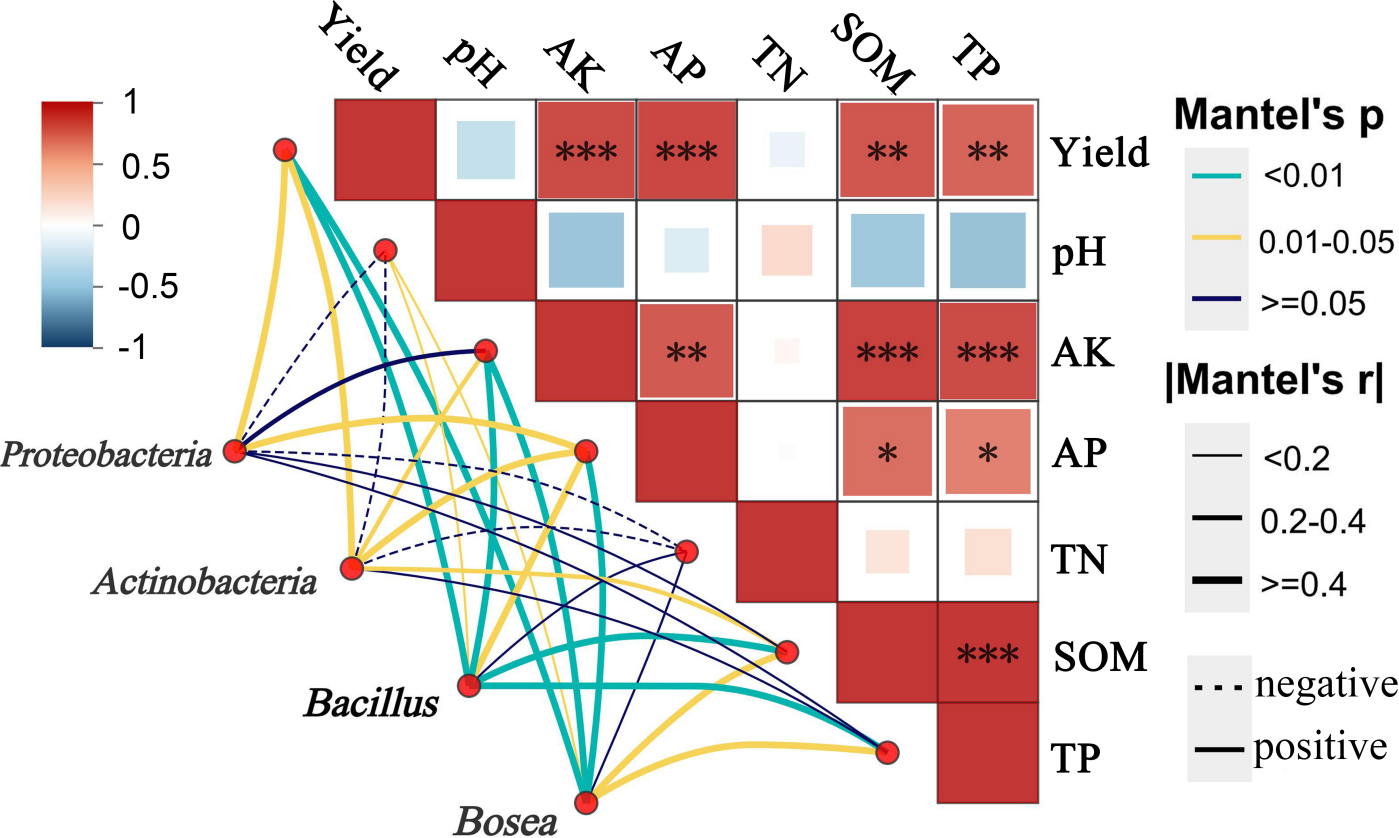
Mantel test correlations between key bacterial taxa (*Proteobacteria, Actinobacteria, Bacillus*, and *Bosea*) and soil properties, including pH, available potassium (AK), available phosphorus (AP), total nitrogen (TN), total phosphorus (TP), soil organic matter (SOM). Edge styles indicate correlation direction (solid meas positive, dashed means negative), and Mantel’s r and *P*-values are shown *means the correlations of significance at (P<0.05); ** (P<0.01); *** (P<0.001).

The original version of this article has been updated.

