# Correction: Bio-organic fertilizers modulate the rhizosphere bacterial community to improve plant yield in reclaimed soil

**DOI:** 10.3389/fpls.2026.1834868

**Published:** 2026-05-04

**Authors:** Cece Qiao, Jing Yang, Qingqin Shao, Jingli Fu, Xueyun Zheng, Jianrong Zhao, Lantian Ren, Wenge Wu, Jianfei Wang

**Affiliations:** 1 College of Resource and Environment, Anhui Science and Technology University/Anhui Engineering Research Center for Smart Crop Planting and Processing Technology, Chuzhou, China; 2 Rice Research Institute, Anhui Academy of Agricultural Sciences, Hefei, China

**Keywords:** reclaimed soil, bio-organic fertilizer, microbial community, plant yield, MiSeq platform 1

The **Author contributions** statement was erroneously given as: “CQ: Investigation, Writing - original draft, Conceptualization. JY: Writing - original draft, Data curation. JF: Investigation, Writing - review & editing, Formal Analysis. XZ: Resources, Formal Analysis, Writing - review & editing. JZ: Software, Investigation, Writing - review & editing. LR: Methodology, Writing -review & editing, Funding acquisition, Formal Analysis. WW: Investigation, Writing - review & editing. JW: Methodology, Writing - review & editing.”

The correct **Author contributions** statement is: “CQ: Investigation, Writing - original draft, Conceptualization. JY: Writing - original draft, Data curation. QS: Formal analysis, Writing - review & editing. JF: Investigation, Writing - review & editing, Formal analysis. XZ: Resources, Formal analysis, Writing - review & editing. JZ: Software, Investigation, Writing - review & editing. LR: Methodology, Writing - review & editing, Funding acquisition, Formal analysis. WW: Investigation, Writing - review & editing. JW: Methodology, Writing - review & editing.”

Author Jing Yang was erroneously assigned as corresponding author. The correct corresponding author is Lantian Ren.

Author Jing Yang was erroneously omitted as equal contributing author. Cece Qiao and Jing Yang contributed equally to this work and share first authorship.

The original version of this article has been updated.

