# Supplementary material for: Bio-organic fertilizers modulate the rhizosphere bacterial community to improve plant yield in reclaimed soil
Source: Front Plant Sci. 2025 Sep 16;16:1660229. doi: 10.3389/fpls.2025.1660229 (PMC12479494; doi:10.3389/fpls.2025.1660229)
Supplement: Supplementary file 1 [file DataSheet1.docx]

Supporting Information

Dear Editor:

Here is the Supporting Information attached to manuscript entitled “**Bio-organic fertilizers modulate therhizosphere bacterial community to improve plant yield in reclaimed soil**”.

Best regards!
Sincerely yours,

Dr. Lantian Ren

College of Resource and Environment, Anhui Science and Technology University/Anhui Engineering Research Center for Smart Crop Planting and Processing TechnologyDonghua Road 9#, Fengyang 233100, P. R. of China. E-mail:1049642413@qq.com, Tel: (86) 0550-6732656.

**Contents:**

Schematic diagram of the rice field experiment design.

Comparison of metabolite expression levels under different fertilization treatments.

The number of quality filtered bacterial 16S rRNA gene sequences and total OTUs

**Fig. S1** Schematic diagram of the rice field experiment design.

**Fig. S2** Comparison of metabolite expression levels under different fertilization treatments.

**Fig. S3** Rarefaction curves of bacterial 16S rRNA sequences at 97% similarity level of soil samples

**Table. S1** The number of quality filtered bacterial 16S rRNA gene sequences and total OTUs

**Figure S1.** Schematic diagram of the rice field experiment design. The experiment included three fertilizer treatments: Chemical fertilizer (CF), Organic fertilizer (OF), and Bio-organic fertilizer (BOF). Each treatment consisted of four plots, with each plot containing 36 seedlings. After transplanting, the crops were observed for 124 days. The study included rice yield estimation, rhizosphere soil sampling, and 16S rRNA gene amplicon sequencing (n = 3) for microbial community analysis.

**Figure S2.** Comparison of metabolite expression levels under different fertilization treatments. The expression levels of metabolites, including Tropolone, p-Toluquinone, Benzoic acid, and others, were measured in soils treated with Chemical Fertilizer (CF), Organic Fertilizer (OF), and Bio-organic Fertilizer (BOF). The results show significant differences in metabolite profiles between treatments, with BOF showing the highest expression levels for most metabolites, followed by OF and CF.

**Fig. S3** Rarefaction curves of bacterial 16S rRNA sequences at 97% similarity level of soil samples

**Table.S1** The number of quality filtered bacterial 16S rRNA genesequences and total OTUs

| soil samples | (OTU numbers) |
| --- | --- |
| CF-1 | 55119 |
| CF-2 | 53770 |
| CF-3 | 50619 |
| CF-4 | 45062 |
| CF-5 | 50994 |
| CF-6 | 23036 |
| OF-1 | 66988 |
| OF-2 | 21012 |
| OF-3 | 44808 |
| OF-4 | 67390 |
| OF-5 | 47051 |
| OF-6 | 51421 |
| BOF-1 | 52574 |
| BOF-2 | 25958 |
| BOF-3 | 35126 |
| BOF-4 | 33777 |
| BOF-5 | 41048 |
| BOF-6 | 45000 |
